# The Queen's Hospital for Children

**Published:** 1909-12-18

**Authors:** J. E. Watts-Ditchfield, C. E. Fox, T. E. Young, J. E. Harwood, Alderman J. R. Brough, H. V. S. Eck, E. R. Ford, Edward S. Burrows, F. W. Goodban, C. Fleming Williams, William J. Davies, G. Isaacs

**Affiliations:** Vicar of St. James-the-Less, Chairman of Emergency Appeal Committee; St. James-the-Less Vicarage, Bethnal Green, E.; Mayor of Bethnal Green; St. James-the-Less Vicarage, Bethnal Green, E.; Mayor of Hackney; St. James-the-Less Vicarage, Bethnal Green, E.; Mayor of Shoreditch; St. James-the-Less Vicarage, Bethnal Green, E.; C.C., Mayor of Stoke Newington; St. James-the-Less Vicarage, Bethnal Green, E.; Rector of Bethnal Green, and Rural Dean; St. James-the-Less Vicarage, Bethnal Green, E.; Vicar and Rural Dean of Shoreditch; St. James-the-Less Vicarage, Bethnal Green, E.; Vicar of St. Stephen's, Haggerston, Hon. Chaplain of Queen's Hospital for Children; St. James-the-Less Vicarage, Bethnal Green, E.; Vicar of St. Chad's, Haggerston, N.E.; St. James-the-Less Vicarage, Bethnal Green, E.; Rectory Road Church, Stoke Newington; St. James-the-Less Vicarage, Bethnal Green, E.; Rector of St. John the Baptist's, Hackney, N.E.; St. James-the-Less Vicarage, Bethnal Green, E.; Minister, South Hackney Synagogue. St. James-the-Less Vicarage, Bethnal Green, E.


### THE QUEEN'S HOSPITAL FOR CHILDREN.

Sir,—At a meeting held to discuss the very serious position of the Queen's Hospital for Children, and attended by the undersigned, and the clergy and ministers of all denominations of the surrounding poverty-stricken districts, it was decided to ask you to emphasise our conviction that the proposed closing, owing to lack of funds, of half the beds of this great children's hospital would be nothing less than a calamity, more especially so at this season of the year.

Anxious as we are to avoid partiality for one hospital when all are doing such excellent work and are so greatly in need of further help, yet we nevertheless strongly feel that this hospital, being one for children, and they of the poorest class, together with the fact that the crisis in its affairs is so grave, the time so short to meet it, the sum required so large, demands from us special consideration at this time.

Knowing the poverty of the district as we do, and realising what it means to the children to suffer illness in their homes—often, alas! used also as the workshop of the family—we would urgently plead that the appalling consequences subsequent to the closing of half the hospital may be averted by generous contributions towards the large sum of £4,000 being forthwith sent to aid a work concerning which we cannot speak in too high praise, and the interruption of which would mean pain and suffering to a class which appeals to all our hearts, the children of the poor.

The amount must be raised before the 31st inst., and we hope that a ready response may be made to our united appeal on behalf of this great work.

Cheques should be sent to the Secretary, Mr. T. Glenton-Kerr, at the Hospital, Hackney Road, Bethnal Green, and should be made payable to him and crossed "Barclay & Co., a/c Queen's Hospital."

We are, Sir, your obedient servants,

J. E. Watts-Ditchfield, Vicar of St. James-the-Less, Chairman of Emergency Appeal Committee; C. E. Fox, Mayor of Bethnal Green; T. E. Young, Mayor of Hackney; J. E. Harwood, Mayor of Shoreditch; Alderman J. R. Brough, C.C., Mayor of Stoke Newington; H. V. S. Eck, Rector of Bethnal Green, and Rural Dean; E. R. Ford, Vicar and Rural Dean of Shoreditch; Edward S. Burrows, Vicar of St. Stephen's, Haggerston, Hon. Chaplain of Queen's Hospital for Children; F. W. Goodban, Vicar of St. Chad's, Haggerston, N.E.; C. Fleming Williams, Rectory Road Church, Stoke Newington; William J. Davies, Rector of St. John the Baptist's, Hackney, N.E.; G. Isaacs, Minister, South Hackney Synagogue.

St. James-the-Less Vicarage, Bethnal Green, E.

December 14, 1909.

